# Specific gut microbiota alterations in essential tremor and its difference from Parkinson’s disease

**DOI:** 10.1038/s41531-022-00359-y

**Published:** 2022-08-05

**Authors:** Pingchen Zhang, Pei Huang, Juanjuan Du, Yixi He, Jin Liu, Guiying He, Shishuang Cui, Weishan Zhang, Gen Li, Shengdi Chen

**Affiliations:** 1 Department of Neurology and Institute of Neurology, Ruijin Hospital, Shanghai Jiao Tong University School of Medicine, Shanghai, 200025 People’s Republic of China; 2 Lab for Translational Research of Neurodegenerative Diseases, Shanghai Institute for Advanced Immunochemical Studies (SIAIS), Shanghai Tech University, Shanghai, 201210 People’s Republic of China

**Keywords:** Diagnostic markers, Parkinson's disease, Risk factors

## Abstract

Essential tremor (ET) is the most common movement disorder and share overlapping symptoms with Parkinson’s disease (PD), making differential diagnosis challenging. Gut dysbiosis is regarded crucial in the pathogenesis of PD. Since ET patients also has comorbidity in gastrointestinal disorders, the relationship between gut microbiota and ET really worth investigating and may help distinguishing ET from PD. Fecal samples from 54 ET, 67 de novo PD and 54 normal controls (NC) were collected for 16S ribosomal RNA gene sequencing and quantitative real-time PCR. ET showed lower species richness (Chao1 index) than NC and PD. ET was with *Bacteroides*-dominant enterotype, while PD was with *Ruminococcus*-dominant enterotype. Compared with NC, 7 genera were significantly reduced in ET, 4 of which (*Ruminococcus, Romboutsia, Mucispirillum,* and *Aeromonas*) were identified to be distinctive with an area under the curve (AUC) of 0.705. Compared to PD, 26 genera were found significantly different from ET, 4 of which (*Bacteroides*, *Fusobacterium*, *Phascolarctobacterium,* and *Lachnospira)* were found distinguishable with an AUC of 0.756. Clinical association results indicated that *Proteus* was associated with disease severity (TETRAS) of ET, while *Klebsiella* was linked to depression and anxiety in ET. Functional predictions revealed that 4 Kyoto Encyclopedia of Genes and Genomes (KEGG) pathways were altered in ET. This study reveals gut dysbiosis in ET and it provides new insight into the pathogenesis of ET and helps distinguishing ET from PD.

## Introduction

Essential tremor (ET) is the most common movement disorder, with a prevalence of more than 60 million individuals worldwide^1^. ET is mainly characterized by an action tremor of the upper limbs, possibly with the involvement of other parts of body, like the head, voice and lower limbs^2^. The clinical profile of ET represented not only with motor symptoms, but also with some non-motor features, including gastrointestinal disorders^3^.

Recently, the crucial role of gut microbiome has been suggested in the pathogenesis of neurodegenerative diseases, such as Parkinson’s disease (PD)^4^, Alzheimer’s disease (AD)^5^, and multiple system atrophy (MSA)^6^. The gut microbiome consists of a large complex community of microbes that colonize the gastrointestinal tract. The brain-gut-microbiome axis not only allows the brain to control gut function but also provides an opportunity for the gut microbiome to influence the brain^7^. An interesting case report of ET showed the symptom improvement after fecal microbiota transplantation^8^, indicating the relationship between gut microbiome and ET. However, the specific microbial alterations of ET have not yet been investigated.

As for gut dysbiosis in neurodegenerative diseases, PD is the most widely studied^9^. The imbalanced microbiome enhanced intestinal permeability and activated the enteric glial cells, which contributed to alpha-synulceinopathy^10^. PD shares certain overlapping features with ET^11^, making it challenging to distinguish one from the other, especially for pseudo-bradykinesia^12^ and sorely action tremor in early-stage PD^13^. On the other hand, some ET patients may develop to PD with the disease progression^14^, making it urgent for us to study the difference between ET and PD in gut dysbiosis.

Thus, our study aimed to find specific gut microbiota alterations in ET and its difference from PD. Previous studies indicated that anti-PD medications, such as catechol-O-methyltransferase (COMT) inhibitors, amantadine and monoamine oxidase B (MAO-B) inhibitors, might interfere with the results of microbiotal alterations analysis^15–17^. Changes of genera *Dorea* and *Phascolarctobacterium* in PD were dose-dependent with levodopa medications^18^. To eliminate the confounding factor of anti-PD medication, we select de novo PD patients as disease control to analyze the gut microbial difference between ET and PD. In addition, since the traditional 16S rRNA sequencing has the shortage of misinterpreting microbial community structures^19^, we adopted quantitative PCR (qPCR) with 16S rRNA gene sequencing data to obtain the accurate estimation of absolute abundance. Moreover, we also included enterotype analysis to see the difference between groups. Finally, we recruited three groups of ET, PD and matched normal controls (NC) to find out the specific gut microbiota changes in ET.

## Results

### Demographics and clinical characteristics of subjects

The demographics and clinical characteristics of ET, NC, and PD were summarized in Table 1. Ten out of the 54 ET patients were treated with beta-blocker (9 with arotinolol and 1 with propranolol) while 3 PD patients and 1 NC took beta-blocker for cardiac concerns. There were no significant difference in age, gender, BMI, MMSE, diabetes, as well as daily habits of smoke, alcohol, tea, coffee among three groups (*P* ≥ 0.085). In addition, ET patients had longer disease duration (*P* < 0.001) and lower SCOPA-AUT total score (*P* = 0.014) compared to PD patients. As for emotion, ET patients exhibited significant higher score in HAMD-17 (*P* < 0.001), and HAMA (*P* < 0.001) compared with NC, but no significant difference was found in HAMD-17 (*P* = 0.297) or HAMA (*P* = 0.840) compared with PD.

**Table 1:** General characteristics of the study subjects.

Variables	ET (*n* = 54)	NC (*n* = 54)	PD (*n* = 67)	*P*
Age(y)^a^	61.72 ± 10.65	62.11 ± 6.46	64.58 ± 10.43	0.123
Male%(male/female)^b^	42.5% (23/31)	44.4% (24/30)	50.75% (34/33)	0.636
BMI(kg/m^2^)^a^	23.52 ± 2.60	23.64 ± 3.25	23.35 ± 2.57	0.962
Smoke^b^	12.9% (7/47)	22.2% (12/42)	20.90% (14/53)	0.405
Alcohol^b^	22.2% (12/42)	16.6% (9/45)	22.39% (15/52)	0.694
Tea^b^	25.9% (14/40)	27.7% (15/39)	28.36% (19/48)	0.954
Coffee^b^	16.6% (9/45)	12.9% (7/47)	8.96% (6/61)	0.443
Diabetes^b^	11.1% (6/48)	9.2% (5/49)	20.90% (15/52)	0.085
MMSE^a^	28.48 ± 1.63	28.06 ± 1.43	28.37 ± 1.60	0.147
HAMD-17^a^	3.33 ± 3.40	1.07 ± 1.87	4.67 ± 5.80	<0.001***
HAMA^a^	3.78 ± 3.25	1.37 ± 2.07	4.49 ± 5.06	<0.001***
Bristol^a^	3.91 ± 1.03	3.96 ± 0.95	4.45 ± 1.34	0.041*
Wexner^a^	1.67 ± 2.31	1.17 ± 2.01	3.42 ± 3.71	0.001**
Constipation^b^	19.6% (10/44)	7.4% (4/50)	23.88% (16/51)	0.055
Disease duration^a^	8.46 ± 8.45	/	2.49 ± 2.04	<0.001***
SCOPA-AUT^a^	3.83 ± 3.82	/	6.58 ± 5.97	0.014*
FTM^a^	9.89 ± 9.63	/	/	/
TETRAS^a^	14.30 ± 9.90	/	/	/
H-Y stage(1.0/1.5/2.0/2.5)^b^	/	/	24/10/23/8	/
MDS-UPDRS^a^	/	/	35.75 ± 20.94	/

*MDS-UPDRS* Movement Disorder Society sponsored version of the Unified Parkinson’s Disease Rating Scale, *MMSE* Mini Mental State Examination, *HAMD-17* Hamilton Depression Scale-17 items, *HAMA* Hamilton Anxiety Scale, *H-Y* Hoehn and Yahr stage, *SCOPA-AUT* Scale for Outcomes in Parkinson’s disease for Autonomic Symptoms, *FTM* Fahn-Tolosa-Marin Clinical Rating Scale for Tremor, *TETRAS* Tremor Research Group (TRG) Essential Tremor Rating Assessment Scale. *P < 0.05, **P < 0.01, ***P < 0.001.

^a^Data were shown as mean ± SD, compared by Kruskal–Wallis/Wilcoxon rank-sum test.

^b^Data were shown as percentage% (number), compared by chi-square/Fisher’s test.

### 16S rRNA sequencing and qPCR results overview

In total, 8,184,232 high-quality 16S rRNA gene V3–V4 sequences were obtained, with 40182 per sample after demultiplexing and quality control. A total of 19 phyla, 27 classes, 49 orders, 80 families, 225 genera, and 321 species were identified. The dominant genera were *Bacteroides* (18.8%), *Prevotella* (5.1%), *Ruminococcus* (3.5%), *Faecalibacterium* (3.3%) in relative abundance data, and *Bacteroides* (2.8 × 10^9^), *Prevotella* (7.4 × 10^8^), *Faecalibacterium* (3.3 × 10^8^) and *Subdoligranulum* (2.6 × 10^8^) in absolute abundance data (Supplementary Fig. 1).

### Microbiota diversity and enterotype distribution among ET, NC and PD

**Fig. 1:**
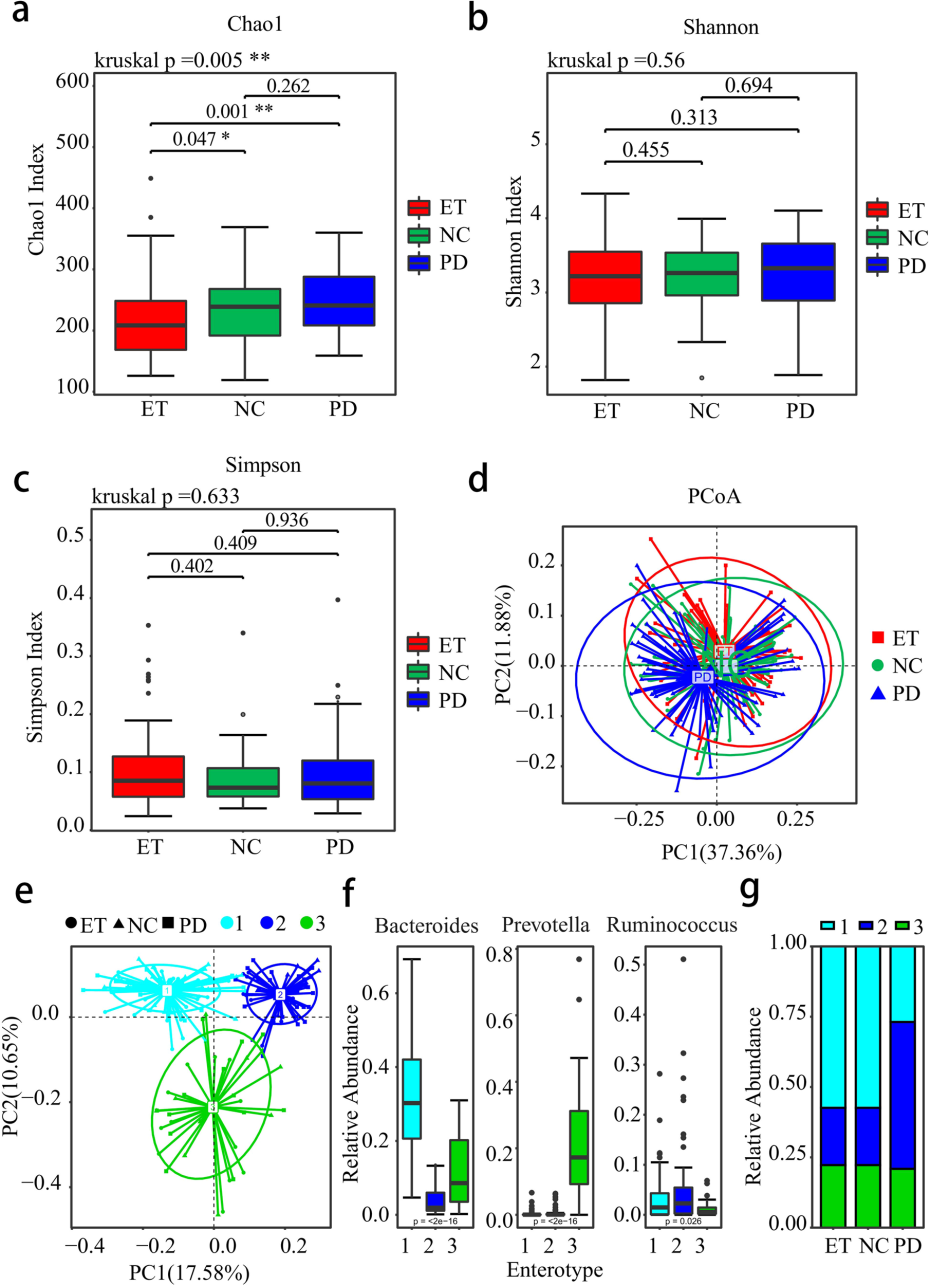
Differences in alpha diversity, beta diversity, and the composition and distribution of enterotype summarized among three groups comparison. **a**–**c** Box plots showing the between-group comparisons of the alpha diversity of gut microbiota by Chao, Simpson, and Shannon index. **d** Beta diversity visualized by PCoA. **e** All fecal samples were clustered into 3 enterotypes. **f** Every color represented the dominant component of each enterotype as light blue standed for *Bacteroides*-dominant enterotype, green standed for *Prevotella*-dominant enterotype and dark blue standed for *Ruminococcus*-dominant enterotype. **g** Three-type enterotype distribution in ET, NC, and PD.

For alpha-diversity, Chao1 index was significantly different among ET, NC and PD groups (Fig. 1a, *P* = 0.005) and Chao1 index of ET patients was lower than that of NC (*P* = 0.047) and PD patients (*P* = 0.001). The significant difference of Chao1 index retained after ridge regression analysis adjusted for the significantly different clinical characteristics (HAMD-17, HAMA, Bristol and Wexner, *P* = 0.016). No significant difference was found in Shannon or Simpson indices among these groups (Fig. 1b, c, *P* = 0.560, *P* = 0.633 respectively).

Additionally, beta-diversity (Weighted Unifrac) was found to be remarkably different among three groups (Fig. 1d) (ANOSIM *R*^2^ = 0.021, *P* = 0.034), especially between ET and PD patients (ANOSIM *R*^2^ = 0.045, *P* = 0.012). However, there is no significant difference between ET and NC (ANOSIM *R*^2^ < 0.001, *P* = 0.868). To determine whether this significance was driven by these four significantly different clinical characteristics (HAMD-17, HAMA, Bristol, and Wexner), PERMANOVA with adjustments showed that the significance of group status retained (*P* = 0.003) and explained 3.4% of the total variation in the microbiome, while Bristol also showed significance (*P* = 0.003) with 2.6% of the total variation.

We next investigated the microbial enterotype features by dividing into 3 clusters (Fig. 1e). Enterotype 1 was dominated by *Bacteroides* as the most enriched genus and *Prevotella* was core in enterotype 2, while *Ruminoccocus* was the predominance in enterotype 3 (Fig. 1f). Significant distribution difference was observed among three groups (*P* < 0.001, Fisher’s test). Difference between ET patients and NC was not significant (*P* = 0.850, Fisher’s test), while comparison between ET and PD showed statistical difference (*P* < 0.001, Fisher’s test).

### Specific fecal microbiota changes in ET compared to NC and PD

**Fig. 2:**
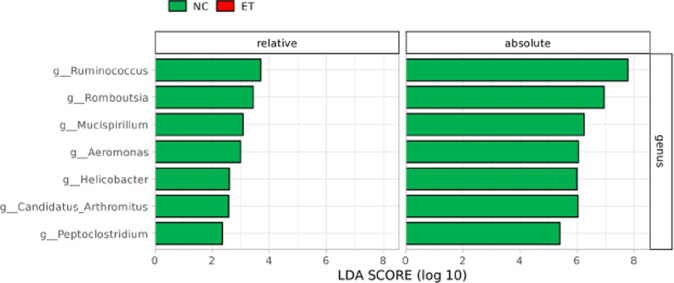
Significant gut microbiota differences between ET and NC in both relative and absolute taxon at genus level. Taxa listed according to their LDA values (superior to 2.0) determined from comparisons between ET and NC groups and only the consistent significant taxa from both relative and absolute data were extracted. LDA linear discriminant analysis.

LEfSE analysis showed key taxa for distinguishing ET patients from NC. Considering the consistent results of relative and absolute data at genus level, ET patients had lower abundances of 7 genera (*Ruminococcus, Romboutsia, Mucispirillum*, *Aeromonas*, *Helicobacter*, *Candidatus Arthromitus* and *Peptoclostridium*) compared to NC (Fig. 2). These genera remained significance after ridge regression adjusted for the statistically different clinical features between ET and NC (HAMD-17 and HAMA). ROC curve from the 4 most distinctive genera (*Ruminococcus, Romboutsia, Mucispirillum,* and *Aeromonas*) selected from their combined relative and absolute abundance data was with an AUC of 0.705 (95%CI 0.608–0.802, sensitivity 64.8% and specificity 64.8%, *P* < 0.001). (Supplementary Fig. 2a)

In particular, only the genus *Peptoclostridium* was found distinctly lower in ET patients whenever compared with NC or PD. Moreover, the abundance of other 17 genera (*Holdemanella*, *Eggerthella*, *Catenibacterium*, *Fastidiosipila*, *Senegalimassilia*, *Chryseobacterium*, *Bacillus*, *Cellulosimicrobium, Anaerococcus*, *Mogibacterium*, *Herbaspirillum*, *Gordonibacter*, *Shuttleworthia*, *Paenibacillus*, *Paracoccus*, *Parascardovia*, *Anaerofustis*) in ET patients was lower than those in PD patients, whereas 8 genera (*Bacteroides*, *Fusobacterium*, *Phascolarctobacterium*, *Lachnospira*, *Lachnoclostridium*, *Bilophila*, *Sutterella*, *Rikenella*) were higher than those in PD patients (Supplementary Fig. 3). Among them, *Cellulosimicrobium* was the only genus affected by Wexner (relative, *P* = 0.040; absolute*, P* = 0.033) in both relative and absolute abundance after ridge regression analysis adjusted for the statistically significant confounding factors (Bristol, Wexner, disease duration and SCOPA-AUT). ROC curve from the 4 most distinctive genera (*Bacteroides*, *Fusobacterium*, *Phascolarctobacterium* and *Lachnospira)* selected from their combined relative and absolute abundance data for the differentiation between ET and PD was with an AUC of 0.756 (95%CI 0.672–0.841, sensitivity 68.6% and specificity 74.0%, *P* < 0.001) (Supplementary Fig. 2b). Besides, in the comparison between PD and NC, the abundance of other 17 genera in PD patients was lower than those in NC, whereas 13 genera were higher in NC than those in PD patients, which was displayed in detail in Supplementary Fig. 4.

### Functional Prediction of fecal microbiota in ET

**Fig. 3:**
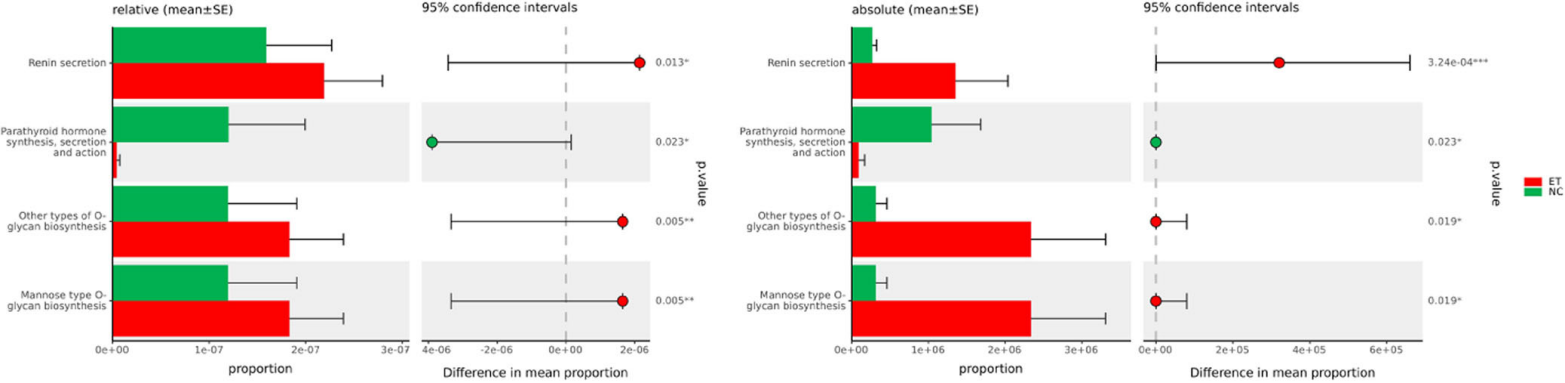
Significant KEGG pathways at level 3 in both relative and absolute data for the fecal microbiome of ET and NC visualized by STAMP. *P < 0.05, **P < 0.01, ***P <0.001.

PICRUSt2 was applied to predict the changes of functional gene composition of fecal microbiota in the samples at KEGG database. There was no statistical difference between ET and NC at level 1 or level 2. At level 3, ET microbiota were enriched for pathways related to renin secretion, mannose type-O-glycan biosynthesis and other types of O-glycan biosynthesis than those of NC, while NC microbiota were enriched for parathyroid hormone synthesis, secretion, and action pathway (Fig. 3). Differences in many functional pathways between ET and PD patients were found. Compared with PD, a total of 68 metabolic pathways (KEGG level 3) were found to be significantly different (*P* < 0.05), including those involved in metabolism of glycan biosynthesis, vitamins, and amino acids, as well as signaling, transport and catabolism. These results were detailed in Supplementary Fig. 5. All these significantly different pathways survived after FDR correction, regardless of the comparison between ET and NC or between ET and PD (Supplementary Tables 1, 2).

### Association between fecal microbiota and clinical features in ET

The associations between fecal microbiota at genus level and clinical features were shown in Supplementary Fig. 6. With the combination of both relative and absolute abundance data of ET, 62 significant paired genus-clinical characteristic correlations were found. (Supplementary Fig. 6). After ridge regression analysis adjusted for confounding factors, 5 significant associations remained: *Klebsiella* had significantly positive correlation with HAMD-17 and HAMD, *Proteus* was positively correlated to HAMD-17 and TETRAS, *Anaerococcus* was negatively linked to MMSE both in relative and absolute abundance data (Table 2 and Supplementary Fig. 7). Besides, when focusing on the 7 distinctive genera between ET and NC identified in LEfSE analysis, we found that *Romboutsia* had significantly positive correlation with Bristol (*r* = 0.30 and *P* = 0.025 in relative abundance, *r* = 0.28 and *P* = 0.038 in absolute abundance), and *Helicobacter* had significantly negative correlation with SCOPA-AUT (*r* = −0.35 and *P* = 0.010 in both relative and absolute abundance). But these above associations did not remain after ridge regression analysis adjusted for confounding factors.

**Table 2:** Spearman’s correlation for fecal microbiota at genus level and clinical features.

		Relative correlation		Absolute correlation	
Genus	Clinical features	coefficient	*P* value	coefficient	*P* value
*Klebsiella*	HAMD-17	0.55	<0.001***	0.49	<0.001***
*Klebsiella*	HAMA	0.31	0.024*	0.27	0.047*
*Proteus*	HAMD-17	0.33	0.014*	0.33	0.015*
*Proteus*	TETRAS	0.39	0.004**	0.39	0.004**
*Anaerococcus*	MMSE	−0.28	0.041*	−0.28	0.043*

All these results were selected from the confirmation of ridge regression analysis. *MMSE* Mini Mental State Examination, *HAMD-17* Hamilton Depression Scale-17 items, *HAMA* Hamilton Anxiety Scale, *TETRAS* Tremor Research Group (TRG) Essential Tremor Rating Assessment Scale. *P < 0.05, **P < 0.01.

## Discussion

Our study analyzed the fecal microbiota alterations in ET and its difference from PD. De novo PD patients were chosen for disease control to exclude the confounding effect of anti-PD medication. To note, 10 of 54 ET patients took beta-blocker as treatment, which was reported to have almost little association with gut microbiota^20^. Therefore, we did not include beta-blocker into the confounding factor for adjustment. Besides, our study also introduced absolute quantification analysis of fecal microbiome except for relative quantification analysis. Finally, the specific gut microbiota changes in ET were performed with functional prediction and clinical association analysis for deep insight into the clinical significance of the findings.

For the bacterial community, our results of alpha diversity indicated that the species richness (Chao1 index) was statistically different in ET patients compared with NC and PD. Beta diversity of Weighted Unifrac distance metrics with adjustments indicated significant difference in three groups’ comparison and the associated clinical feature (Bristol) might affect the gut microbiome. In particular, the enterotype profiling results between the three groups showed significant difference. Enterotype profiling refers to the stratification of human gut microbiota and is regarded as a reliable method to understand the microbiota community irrespective of age, gender, and ethnicity^21^. In our study, we adopted the classical three-enterotype model^22^ and found that the ET was mostly *Bacteroides*-dominant, similar to the distribution of NC. Whereas PD was with a tendency towards the enterotype dominated by *Ruminococcus*. *Ruminococcus*-dominant enterotype was reported to have the lowest overall bacterial growth rate^23^, possibly indicating the fragility to protect the equilibrium of intestinal barrier and the aggravation of intestinal inflammation in PD pathogenesis. Although the enterotype itself might not be sufficiently useful for differentiating ET and PD, it may still be relevant in various clinical settings, including potential disease association^24^.

Regardless of the similarity of enterotype distribution between ET and NC, there still existed taxonomic differences. At genus level, there were reduced abundances of 7 genera in ET patients than those in NC, including *Ruminococcus, Romboutsia, Mucispirillum*, *Aeromonas*, *Helicobacter*, *Candidatus Arthromitus,* and *Peptoclostridium*, not influenced by differential clinical characteristics. *Romboutsia* and *Ruminococcus* are both fermenting anaerobes that lead to the production of short-chain fatty acids (SCFAs)^25,26^. SCFAs are the provital part to maintain the stability of colonic mucosa and fight against intestinal inflammation^27^. In addition, *Candidatus Arthromitus* was proved to be protective for healthy gut and could help fighting against pathogens resulting in morbidity and decreased performance in turkey model^28^. The decrease of these beneficial genera may participate in the immune response of opportunistic pathogens and the modulation of intestinal permeability. Besides, *Helicobacter*^29^, *Peptoclostridium*^30^ and *Aeromonas*^31^ were considered multifactorial and could lead to the immune host response in certain virulent circumstances. Although we couldn’t clarify the precise role of the altered gut microbiota in ET due to its limitation to species level interpretation, we still confirmed its specific taxa difference and this published literature of such microbial function could give hints for further research.

The gut microbiota changes in ET compared with PD provided the first evidence of the difference in gut dysbiosis of ET from PD. Of which, some taxa were reported in the previous studies of PD gut dysbiosis compared with NC, such as increased *Eggerthella*^26^ and *Peptoclostridum*^32^, decreased *Bacteroides*^33^ in PD patients and these aforementioned alterations were the same as in the comparison between PD and NC in our study. This finding indicated that some taxa alterations might be specific for PD. Besides, the comparison between PD and NC based on de novo cases and quantitative approach provided more robust evidences for the gut dysbiosis in PD without the interference of anti-PD medication. In addition to the 3 genus (increased *Eggerthella*^26^ and *Peptoclostridum*^32^, and decreased *Bacteroides*^33^*)* mentioned above, increased *Catabacter*^34^ and decreased *Faecalibacterium*^34^ presented the same trend with previous study while the other novel findings (like increased *Holdemanella*, increased *Chryseobacterium*,etc) needed larger numbers of de novo subjects with the same approach to be confirmed.

Based on the results of between-group comparisons, 4 genera (*Ruminococcus, Romboutsia, Mucispirillum,* and *Aeromonas*) were selected for the distinction of ET from NC with an AUC of 0.705. For differential diagnosis between ET and PD, 4 genera (*Bacteroides*, *Fusobacterium*, *Phascolarctobacterium,* and *Lachnospira)* were found remarkable with an AUC of 0.756. Overall, specific gut microbiota alterations might be a useful indicator to differentiate ET from NC and PD. Further investigation for a broader analysis of microbial composition seemed to be worthwhile.

The comparison of functional prediction between ET and NC indicated 4 altered metabolic pathways according to the KEGG hierarchical level 3 classification, which could give insights to the following research on the interaction between gut dysbiosis and the host. Further study using metagenomic sequencing and metabolites analysis to study the functional alterations of microbes in ET is crucial.

Correlation analysis of specific taxa and clinical symptoms found a positive correlation between *Proteus* and TETRAS. Although no previous study has directly mentioned the association between ET severity and microbiota, an animal study has shown the administration of one specie of *Proteus (Proteus mirabilis)* could induce motor deficits via gut leakage and brain inflammation^35^. On the other hand, *Klebsiella* was found positively related to emotional change in ET, such as anxiety and depression. Previous researches have also confirmed that an increase of *Klebsiella* was associated with major depressive disorder^36^ and could induced anxiety in mice model^37^. In all, these previous studies were consistent with the correlation between gut microbiota and clinical features in our study. Thus, despite that functional studies of gut microbiota need to focus on specie level instead of genus level, our results could still give limited clues for further study.

There are still several limitations in our research. Firstly, given the cross-sectional nature of our study, the observational results cannot prove cause and effect association. Secondly, dietary patterns and food preferences may impact the microbiota expression, a larger-scale research samples from different populations are warranted, and a complete and systematic dietary interrogation, like EPIC-Norfolk Food Frequency Questionnaire^38^, should be added in the future study.

In conclusion, this study provides preliminary data revealing gut dysbiosis in ET, and identify the relation between gut microbiota and clinical features. It also provides new insight into the pathogenesis of ET and help distinguishing ET from PD.

## Methods

### Study subjects

One hundred and seventy-five subjects (54 ET, 67 PD, and 54 NC) from outpatient clinic of Movement disorders Center in Ruijin Hospital affiliated to Shanghai Jiao Tong University School of Medicine were enrolled in our study between January 2018 and August 2021. This study was approved by the Institutional Review Board of Ruijin Hospital affiliated to Shanghai Jiao Tong University School of Medicine. Written informed consents were obtained from all of the participants. Inclusion criteria were: (1) aged 25–85 years, (2) ET patients were diagnosed according to MDS Task Force criteria^2^ and PD diagnosis was based on MDS criteria^39^ for PD patients, (3) no anti-PD medication intake before the fecal sample collection of all subjects, (4) solely beta-blocker intake or no relevant medication intake before the fecal sample collection for ET group. NC matched by age, gender, and body mass index (BMI) were selected simultaneously. Exclusion criteria were: (1) vegetarian or malnutrition, (2) chronic gastrointestinal disorder (including inflammatory bowel disease, gastric or duodenal ulcer), (3) severe chronic illness (including malignant tumor, heart failure, renal insufficiency, hematological disorder, etc), (4) history of major gastrointestinal surgery, (5) ongoing or regular consummation of yogurt, (6) no use of any probiotic or antibiotic within one month, (7) ongoing use of corticosteroid, proton pump inhibitor, statin, Metformin, immunosuppressant or anti-neoplastic medication, (8) severe cognitive deficit that obstructed the execution of clinical assessment.

### Clinical evaluation

All subjects provided information of medical history, weight, and height for the calculation of BMI, and accepted neurological examination and clinical assessment, such as Hamilton Anxiety Scale (HAMA) for anxiety^40^, Hamilton Depression Scale-17 items (HAMD-17) for depression^41^, Wexner constipation score and Bristol stool scale for constipation severity^42,43^, Mini Mental State Examination (MMSE) for cognition^44^. The Scale for Outcomes in Parkinson’s disease for Autonomic Symptoms (SCOPA-AUT) was interrogated for ET and PD patients for autonomic dysfunction^45^. Fahn-Tolosa-Marin Clinical Rating Scale for Tremor (FTM)^46^ and the Tremor Research Group (TRG) Essential Tremor Rating Assessment Scale (TETRAS)^47^ were examined among ET patients; the Movement Disorder Society sponsored version of the Unified Parkinson’s Disease Rating Scale (MDS-UPDRS)^48^ and the Hoehn and Yahr stage (H-Y stage)^49^ were examined for PD patients, respectively.

### Fecal sample collection and sequencing

In accordance with our previous study^6^, each participant was asked to collect a fecal sample in the morning using fecal collection containers. The containers were transferred on ice and stored at −80 °C prior to processing. The DNA was extracted from 200 mg samples using the QIAamp ® Fast DNA Stool Mini Kit (QIAGEN, Hilden, Germany) following the manufacturer’s instructions. Microbial composition was determined by 16S rRNA gene sequencing of DNA extracted from stool by amplifying the V3–V4 regions. DNA was checked by running the samples on 1.2% agarose gels. Polymerase chain reaction (PCR) amplification of 16S rRNA genes was performed using general bacterial primers (357F and 806R) with two-step amplicon library building on the Novaseq platform.

At the same time, we calculated the total bacteria load with 3 replicates by using quantitative real-time PCR. The PCR product of the target microbiota gene was used as a standard control, followed by amplification using the TaKaRa® SYBR PremixTaq kit according to the manufacturers’ instructions. PCR amplification was performed using the same general primers (357F and 806R). The reaction conditions were as follows: pre-denaturation at 95 °C for 30 s, denaturation at 95 °C for 10 s, annealing at 55 °C for 30 s, extension at 72 °C for 30 s (40 cycles), and final extension at 72 °C. Standard curves were set up by serially diluting plasmid of a pMD18-T vector with the appropriate insert from 10^7^ to 10^12^ target gene copies µl^−1^ for each primer set. The details were described in Supplementary Information.

### Statistical analysis

The 16S sequences were analyzed by using a combination of software Trimmomatic^50^ (version 0.35), Flash^51^ (version 1.2.11), UPARSE^52^ (version v8.1.1756), mothur^53^ (version 1.33.3) and R^54^ (version 3.6.3). The raw 16s rRNA gene data were processed to form operational taxonomic units (OTUs) at 97% identity using UPARSE. Taxonomy was assigned using Silva 128 as the reference database. The relative abundances obtained from 16S rRNA sequencing were quantified to get absolute abundance (copies/gram) by multiplying the total bacterial load performed by qPCR.

The characteristics of all subjects were compared by Kruskal–Wallis/Wilcoxon rank-sum test and chi-squared test/Fisher’s exact test.

Alpha and beta diversity were analyzed based on the absolute abundance. Alpha diversity analysis was performed using Chao1 index for species richness, Shannon and Simpson indices for species diversity. The ridge regression analysis was then performed by adjusted for the significantly different clinical characteristics among groups to avoid the confounding factors. Beta diversity analysis between groups was visualized by principal coordinate analysis (PCoA) and calculated by ANOSIM with weighted Unifrac analysis^55^. To control confounding factors, we conducted permutational multivariate analysis of variance (PERMANOVA)^56^ with groups and used those significantly different characteristics as covariates, and the fraction of the total variance explained by each variable was calculated in this model. Meanwhile, enterotype features were examined by dividing into 3 clusters by principal coordinate analysis based on the Jensen-Shannon divergence^22^.

The genus level was selected for further analysis and the following analysis were based on the consistent results of relative and absolute abundance data. Linear discriminant analysis (LDA) Effect Size (LEfSE) analysis^57^ was used for between-group comparisons with an alpha cutoff of 0.05 and an effect size cutoff of 2.0. Also, ridge regression analysis was conducted for controlling the confounding factors. The 4 most discriminant genera were identified by LEfSE analysis between groups with the highest LDA scores extracted from the consistent results (overlapped genera) of relative and absolute data. Subsequently, the data of relative abundance, absolute abundance, and the combination of relative and absolute abundance of the four most discriminant genera were put into the receiver operating characteristics curve analysis, and their areas under the curve (AUC) were obtained, respectively.

Updated Phylogenetic Investigation of Communities by Reconstruction of Unobserved States (PICRUSt2)^58^ was used for functional prediction in Kyoto Encyclopedia of Genes and Genomes (KEGG). The significant *P* value in KEGG was then adjusted by false discovery rate (FDR). Spearman rank-correlation analysis was applied to explore the relationship between clinical features and specific microbiota taxa in ET. The ridge regression analysis was then performed to confirm the correlation effect, adjusting for sex, age, BMI, smoking, alcohol drinking, coffee drinking, tea drinking, and diabetes. *P* < 0.05 was considered as statistically significant.

## Supplementary information

Supplementary Material

## Data Availability

The original 16S and qPCR sequencing data were deposited in the National Center for Biotechnology Information (NCBI) BioProject database (PRJNA822998) with an URL of https://www.ncbi.nlm.nih.gov/Traces/study/?acc=SRP367652&o=acc_s%3Aa. Other relevant data that support the findings of this study are available from the corresponding author upon reasonable request.

## Code availability

Only open source code from a combination of software Trimmomatic (version 0.35), Flash (version 1.2.11), UPARSE (version v8.1.1756), mothur (version 1.33.3) and R (version 3.6.3) were used, using either the default settings or the settings stated in the “Methods” section. Further information for clarification is available from the corresponding author on reasonable request.

## References

[1] Welton T (2021). Essential tremor. Nat. Rev. Dis. Prim..

[2] Bhatia KP (2018). Tremor Task Force of the International Parkinson and Movement Disorder Society. Consensus Statement on the classification of tremors from the task force on tremor of the International Parkinson and Movement Disorder Society. Mov. Disord..

[3] Pradeep S, Mehanna R (2021). Gastrointestinal disorders in hyperkinetic movement disorders and ataxia. Parkinsonism Relat. Disord..

[4] Haikal C, Chen QQ, Li JY (2019). Microbiome changes: An indicator of Parkinson’s disease?. Transl. Neurodegener..

[5] Li B (2019). Mild cognitive impairment has similar alterations as Alzheimer’s disease in gut microbiota. Alzheimers Dement..

[6] Du J (2019). Fecal and blood microbial 16s rRNA gene alterations in Chinese patients with multiple system atrophy and its subtypes. J. Parkinsons Dis..

[7] Schmidt T, Raes J, Bork P (2018). The human gut microbiome: From association to modulation. Cell.

[8] Liu XJ, Wu LH, Xie WR, He XX (2020). Faecal microbiota transplantation simultaneously ameliorated patient’s essential tremor and irritable bowel syndrome. Psychogeriatrics.

[9] Dogra N, Mani RJ, Katare DP (2022). The gut-brain axis: Two ways signaling in Parkinson’s disease. Cell Mol. Neurobiol..

[10] Luo S (2021). The pivotal role of microbiota in modulating the neuronal-glial-epithelial unit. Infect. Drug Resist..

[11] Algarni M, Fasano A (2018). The overlap between essential tremor and Parkinson disease. Parkinsonism Relat. Disord..

[12] Paparella G, Fasano A, Hallett M, Berardelli A, Bologna M (2021). Emerging concepts on bradykinesia in non-parkinsonian conditions. Eur. J. Neurol..

[13] Coria F (2012). Nigrostriatal dopaminergic function in subjects with isolated action tremor. Parkinsonism Relat. Disord..

[14] Thenganatt MA, Jankovic J (2016). The relationship between essential tremor and Parkinson’s disease. Parkinsonism Relat. Disord..

[15] Scheperjans F (2015). Gut microbiota are related to Parkinson’s disease and clinical phenotype. Mov. Disord..

[16] Bedarf JR (2017). Functional implications of microbial and viral gut metagenome changes in early stage L-DOPA-naïve Parkinson’s disease patients. Genome Med..

[17] Hill-Burns EM (2017). Parkinson’s disease and Parkinson’s disease medications have distinct signatures of the gut microbiome. Mov. Disord..

[18] Qian Y (2018). Alteration of the fecal microbiota in Chinese patients with Parkinson’s disease. Brain Behav. Immun..

[19] Vandeputte D (2017). Quantitative microbiome profiling links gut community variation to microbial load. Nature.

[20] Jackson MA (2018). Gut microbiota associations with common diseases and prescription medications in a population-based cohort. Nat. Commun..

[21] Cheng M, Ning K (2019). Stereotypes about enterotype: The old and new ideas. Genomics Proteom. Bioinform..

[22] Arumugam M (2011). Enterotypes of the human gut microbiome. Nature.

[23] Vieira-Silva S (2016). Species-function relationships shape ecological properties of the human gut microbiome. Nat. Microbiol..

[24] Costea PI (2018). Enterotypes in the landscape of gut microbial community composition. Nat. Microbiol..

[25] Louis P, Flint HJ (2017). Formation of propionate and butyrate by the human colonic microbiota. Environ. Microbiol..

[26] Gerritsen J (2017). Genomic and functional analysis of *Romboutsia* ilealis CRIBT reveals adaptation to the small intestine. PeerJ.

[27] Dalile B, Van Oudenhove L, Vervliet B, Verbeke K (2019). The role of short-chain fatty acids in microbiota-gut-brain communication. Nat. Rev. Gastroenterol. Hepatol..

[28] Hedblom GA, Reiland HA, Sylte MJ, Johnson TJ, Baumler DJ (2018). Segmented filamentous bacteria—metabolism meets immunity. Front. Microbiol..

[29] Ménard A, Smet A (2019). Review: Other *Helicobacter* species. Helicobacter.

[30] Tougas SR (2021). Prevalence of detection of *Clostridioides difficile* among asymptomatic children: A systematic review and meta-analysis. JAMA Pediatr..

[31] Fernández-Bravo A, Figueras MJ (2020). An Update on the Genus *Aeromonas*: Taxonomy, Epidemiology, and Pathogenicity. Microorganisms.

[32] Li Y (2020). Features of gut microbiota in patients with idiopathic Parkinson’s disease. Zhonghua yi xue za zhi..

[33] Li F (2019). Alteration of the fecal microbiota in North-Eastern Han Chinese population with sporadic Parkinson’s disease. Neurosci. Lett..

[34] Petrov VA (2017). Analysis of gut microbiota in patients with Parkinson’s disease. Bull. Exp. Biol. Med..

[35] Choi JG (2018). Oral administration of *Proteus mirabilis* damages dopaminergic neurons and motor functions in mice. Sci. Rep..

[36] Cheung SG (2019). Systematic review of gut microbiota and major depression. Front. Psychiatry.

[37] Jang HM (2018). Evidence for interplay among antibacterial-induced gut microbiota disturbance, neuro-inflammation, and anxiety in mice. Mucosal Immunol..

[38] Bingham SA (2001). Nutritional methods in the European Prospective Investigation of Cancer in Norfolk. Public Health Nutr..

[39] Postuma RB (2015). MDS clinical diagnostic criteria for Parkinson’s disease. Mov. Disord..

[40] Martinez-Martin P (2016). Accuracy of screening instruments for detection of neuropsychiatric syndromes in Parkinson’s disease. Mov. Disord..

[41] Schrag A (2007). Depression rating scales in Parkinson’s disease: Critique and recommendations. Mov. Disord..

[42] Agachan F, Chen T, Pfeifer J, Reissman P, Wexner SD (1996). A constipation scoring system to simplify evaluation and management of constipated patients. Dis. Colon Rectum..

[43] Blake MR, Raker JM, Whelan K (2016). Validity and reliability of the Bristol Stool Form Scale in healthy adults and patients with diarrhoea-predominant irritable bowel syndrome. Aliment Pharm. Ther..

[44] Skorvanek M (2018). Global scales for cognitive screening in Parkinson’s disease: Critique and recommendations. Mov. Disord..

[45] Visser M, Marinus J, Stiggelbout AM, Van Hilten JJ (2014). Assessment of autonomic dysfunction in Parkinson’s disease: The SCOPA-AUT. Mov. Disord..

[46] Fahn, S., Tolosa, E. & Marin, C. Clinical rating Scale for Tremor. In *Parkinson’s Disease and Movement Disorders*. (eds. Jankovik, J. & Tolosa, E.) 225–234 (Urban & Schwarzenberg, Baltimore-Munich, 1988).

[47] Elble R (2012). Reliability of a new scale for essential tremor. Mov. Disord..

[48] Goetz CG (2007). Movement Disorder Society-sponsored revision of the Unified Parkinson’s Disease Rating Scale (MDS-UPDRS): Process, format, and clinimetric testing plan. Mov. Disord..

[49] Goetz CG (2004). Movement Disorder Society Task Force report on the Hoehn and Yahr staging scale: Status and recommendations. Mov. Disord..

[50] Bolger AM, Lohse M, Usadel B (2014). Trimmomatic: A flexible trimmer for Illumina sequence data. Bioinformatics.

[51] Magoč T, Salzberg SL (2011). FLASH: Fast length adjustment of short reads to improve genome assemblies. Bioinformatics.

[52] Alloui, T. et al. Usearch: A Meta Search Engine based on a new result merging strategy. In *2015 7th International Joint Conference on Knowledge Discovery, Knowledge Engineering and Knowledge Management (IC3K)* 531–536 (2015).

[53] Schloss PD (2009). Introducing mothur: Open-source, platform-independent, community-supported software for describing and comparing microbial communities. Appl. Environ. Microbiol..

[54] Ihaka R, Gentleman RR (1996). A language for data analysis and graphics. J. Comput. Graph Stat..

[55] Clarke KR (1993). Non-parametric multivariate analyses of changes in community structure. Austral Ecol..

[56] Anderson MJ (2001). A new method for non-parametric multivariate analysis of variance. Austral Ecol..

[57] Segata N (2011). Metagenomic biomarker discovery and explanation. Genome Biol..

[58] Douglas, G. M. et al. PICRUSt2: An improved and customizable approach for metagenome inference. 10.1101/672295 (2020).

