# Supplementary material for: Specific gut microbiota alterations in essential tremor and its difference from Parkinson’s disease
Source: NPJ Parkinsons Dis. 2022 Aug 5;8:98. doi: 10.1038/s41531-022-00359-y (PMC9355955; doi:10.1038/s41531-022-00359-y)
Supplement: Supplementary file 1 — Supplementary Material [file 41531_2022_359_MOESM1_ESM.pdf]

Supplementary Files

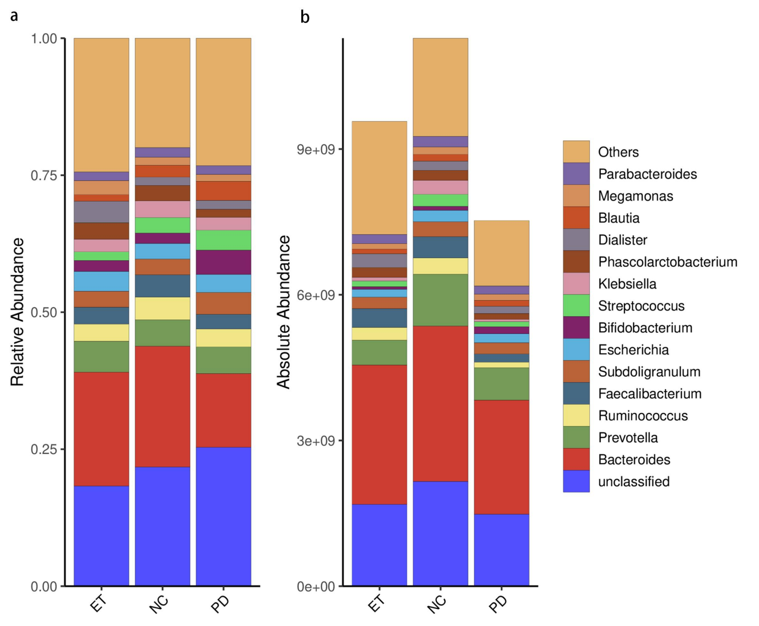

Supplementary Figure 1 Histogram of relative versus absolute microbial distribution in patients with ET, NC and PD at the genus level. a The microbial distribution of relative abundance in ET, NC and PD. **b** The microbial distribution of absolute abundance in ET, NC and PD. The top 15 most abundant genera were depicted, with all others included into "others".

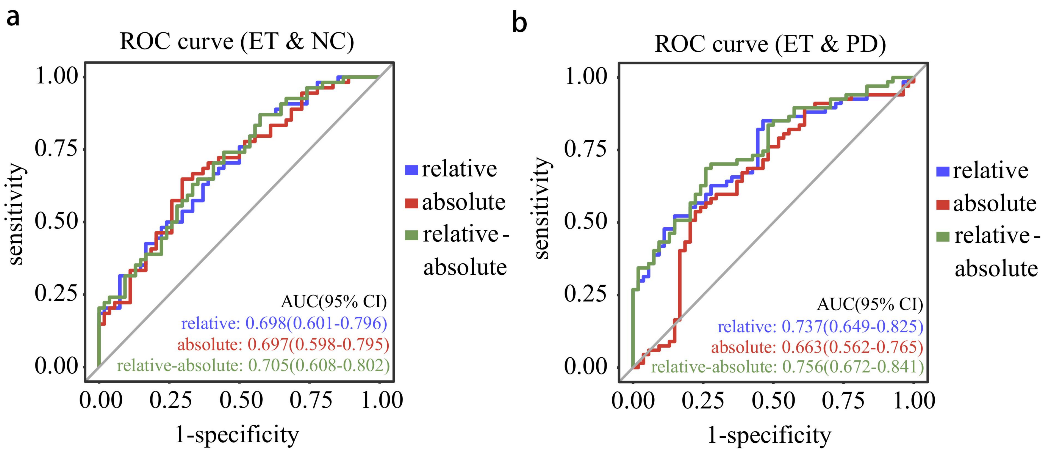

Supplementary Figure 2 ROC curves used for the differential diagnosis by the relative, absolute and the combination of relative& absolute abundance of 4 most discriminant genera with the highest LDA scores between groups. a Combination of *Ruminococcus, Romboutsia, Mucispirillum* and *Aeromonas* identified between ET and NC showed an AUC of 0.705 when combining the consistent results from relative and absolute data. **b** Combination of *Bacteroides*, *Fusobacterium*, *Phascolarctobacterium* and *Lachnospira* identified between patients with ET and PD showed an AUC of 0.756 when combining the consistent results from relative and absolute data.

LDA, linear discriminant analysis, ROC, receiver operating characteristic curve; AUC, area under the curve.

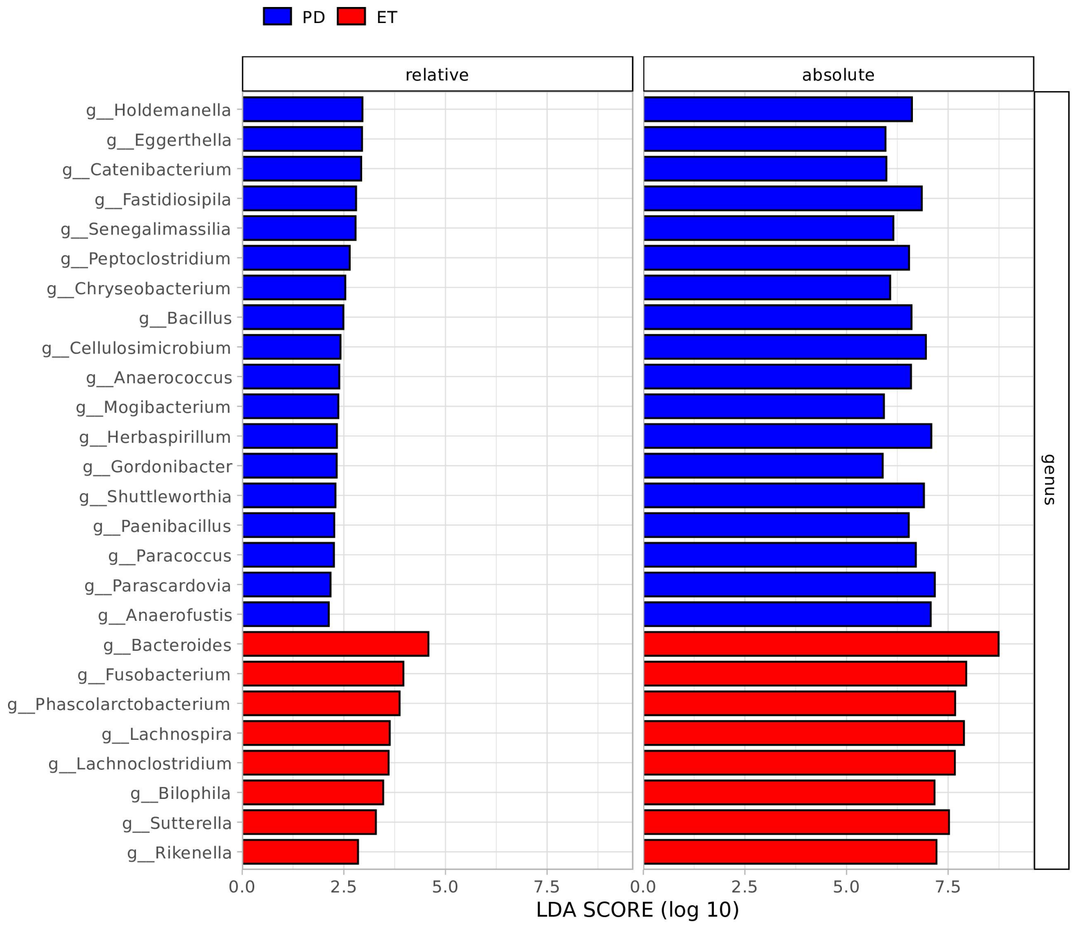

Supplementary Figure 3 Significant gut microbiota differences between PD and ET in both relative and absolute result at genus level.

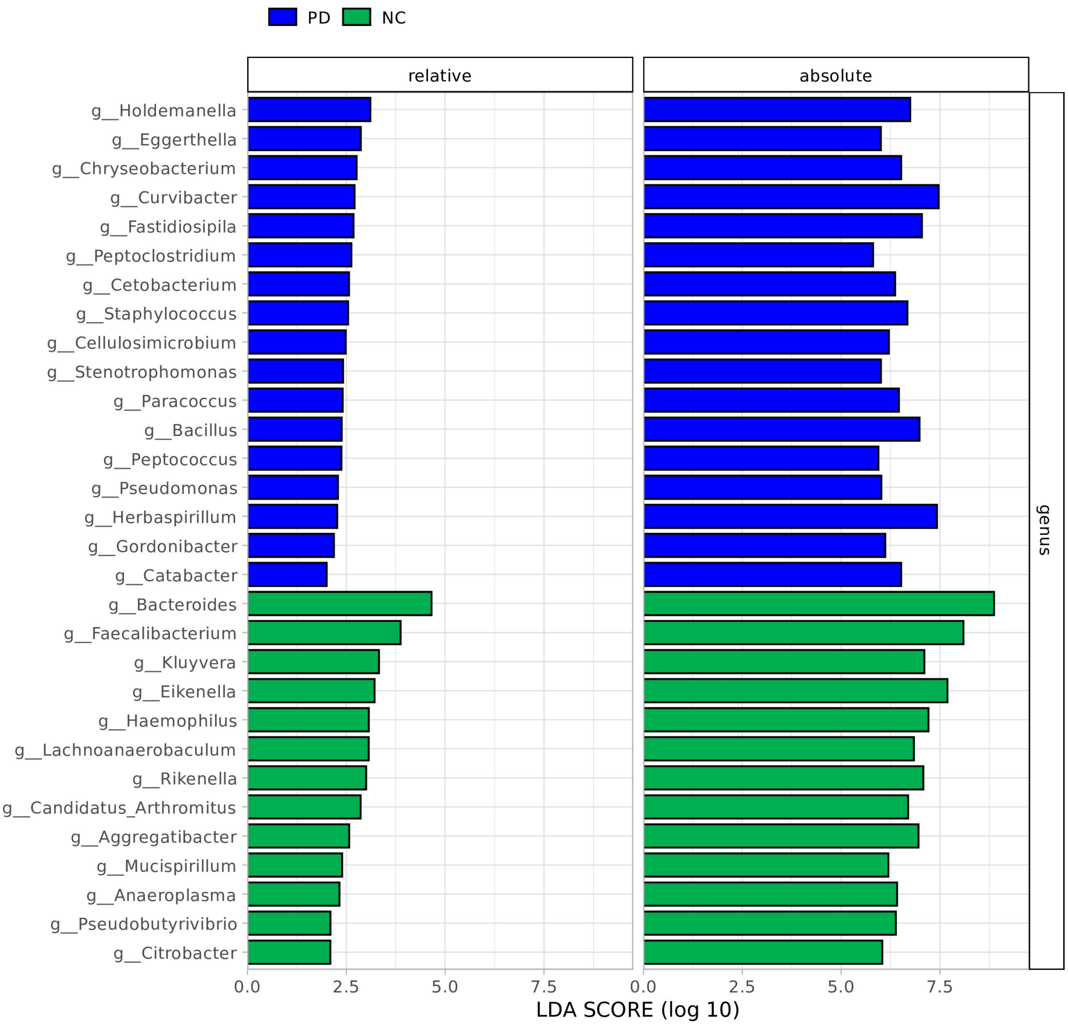

Supplementary Figure 4 Significant gut microbiota differences between PD and NC in both relative and absolute result at genus level.

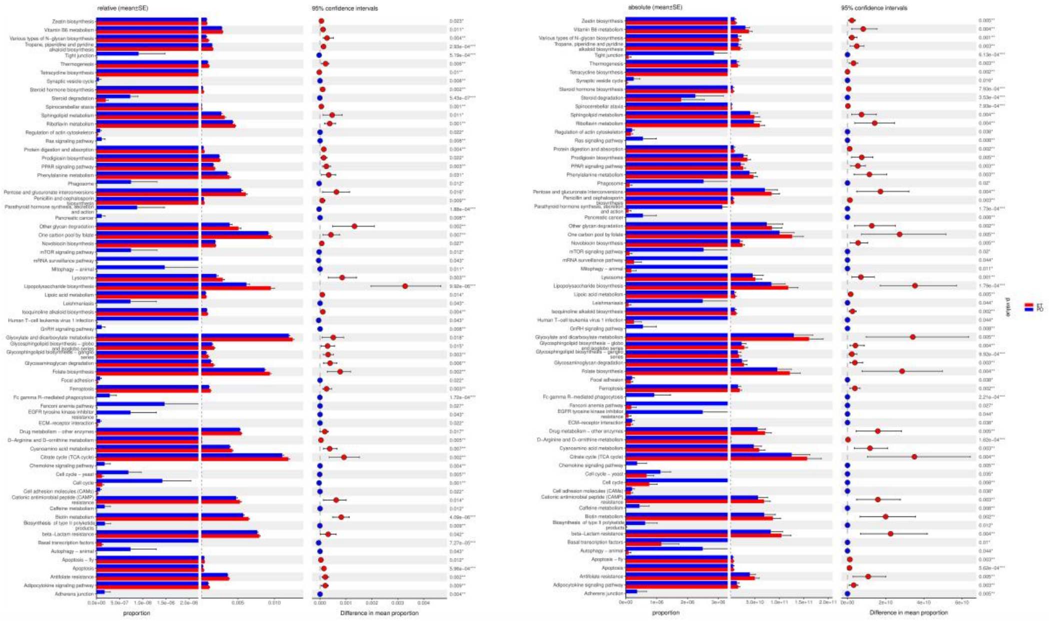

Supplementary Figure 5 Significant KEGG pathways at level 3 in both relative and absolute data for the fecal microbiome of the PD and ET visualized by STAMP.

* P < 0.05, ** P < 0.01, *** P＜0.001.

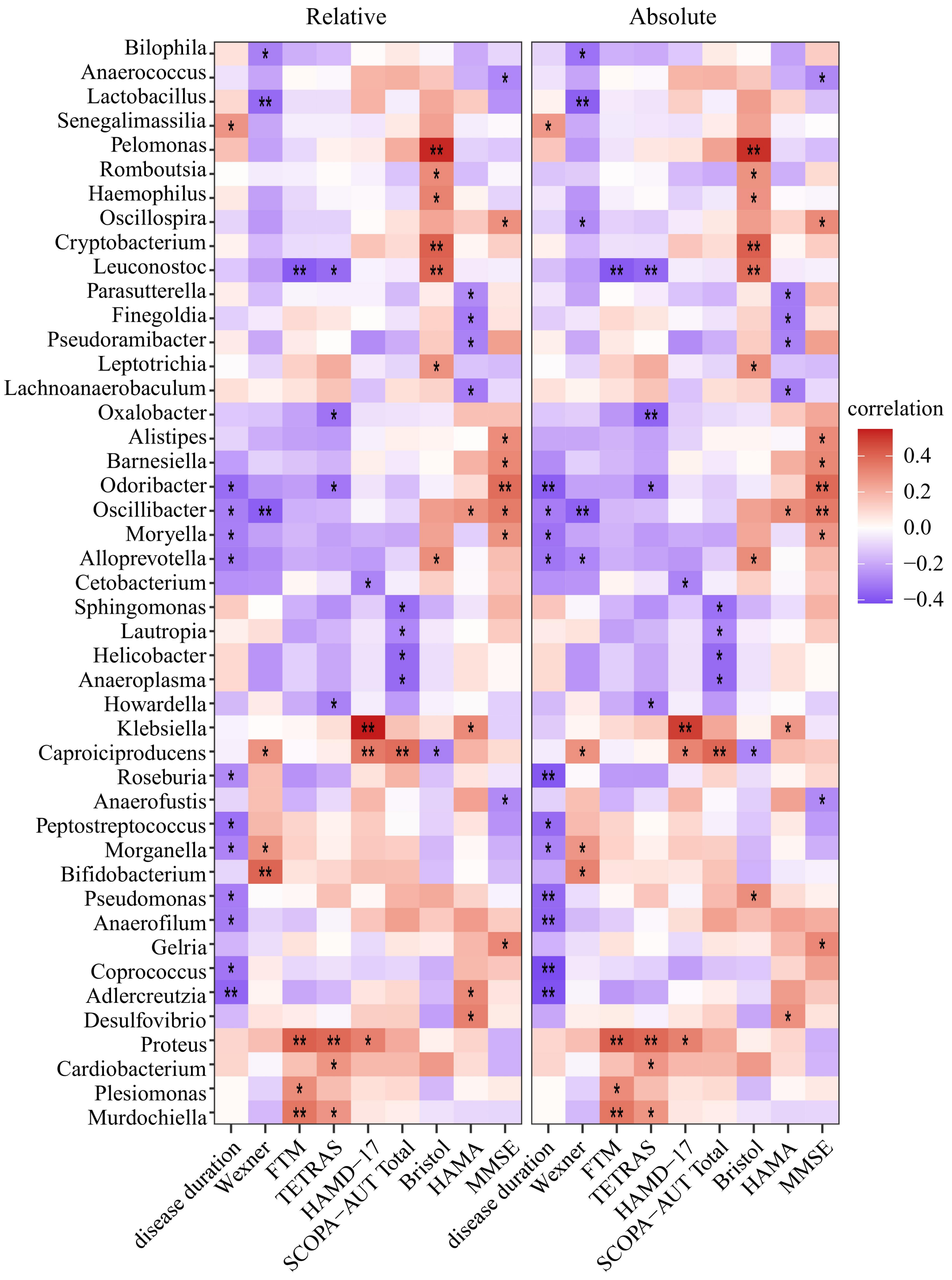

Supplementary Figure 6 Heatmaps showing the associations between clinical features and fecal microbiota at genus level. a The significant association in the relative data **b** The significant association in the absolute data. The intensity of the color represented the “r” value (negative score,

blue; positive score, pink).

MMSE Mini Mental State Examination, *HAMD-17* Hamilton Depression Scale-17 items, *HAMA* Hamilton Anxiety Scale, *SCOPA-AUT* Scale for Outcomes in Parkinson’s disease for Autonomic Symptoms, *FTM* Fahn-Tolosa-Marin Clinical Rating Scale for Tremor, *TETRAS* Tremor Research Group (TRG) Essential Tremor Rating Assessment Scale. Spearman test, * $P < 0.05$, ** $P < 0.01$.

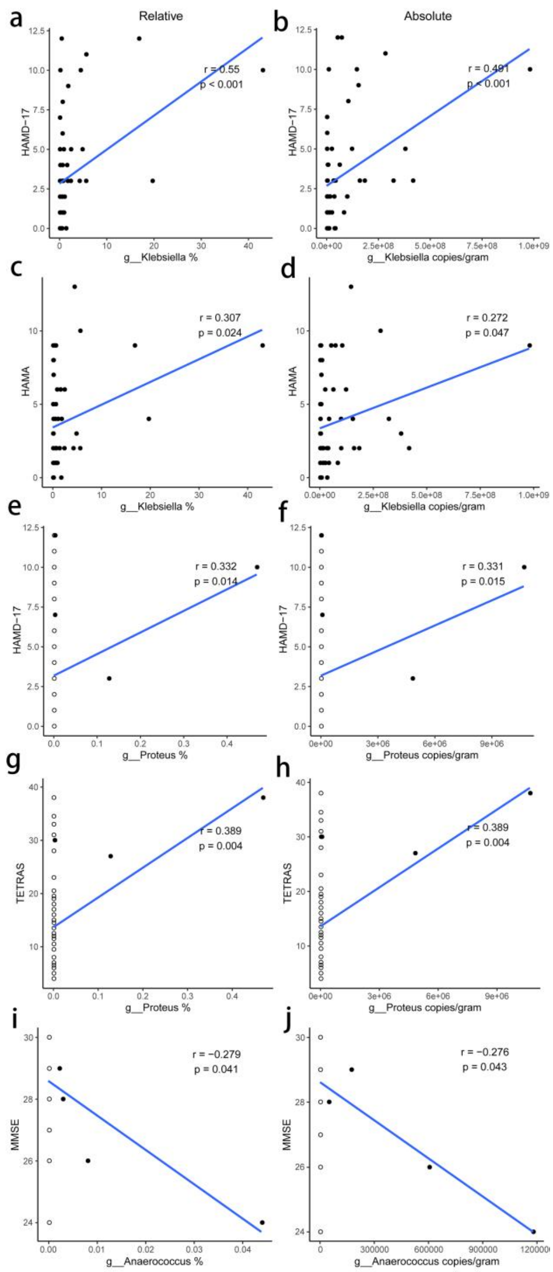

Supplementary Figure 7 Scatter plots showing the association between specific taxon and clinical features in relative and absolute data. a-b The association of *Klebsiella* and HAMD-17. **c-d** The association of *Klebsiella* and HAMA. **e-f** The association of *Proteus* and HAMD-17. **g-h** The association of *Proteus* and TETRAS. **i-j** The association of *Anaerococcus* and MMSE. Solid points represented detected samples and hollow points represented absent samples. *HAMD-17* Hamilton Depression Scale-17 items, *HAMA* Hamilton Anxiety Scale, *TETRAS* Tremor Research Group (TRG) Essential Tremor Rating Assessment Scale.

Supplementary Table 1 Functional differences between ET and NC based on the mean abundances of KEGG pathways at level 3.

**Pathways**	**Relative**				**Absolute**			
	**Mean±SD (ET, n=54)**	**Mean±SD (NC, n=54)**	***P* value**	**FDR corrected *P* value**	**Mean±SD (ET, n=54)**	**Mean±SD (NC, n=54)**	***P* value**	**FDR corrected *P* value**
**Mannose type O-glycan biosynthesis**	1.83×10^{-7} ± 4.11×10^{-7}	1.19×10^{-7} ± 5.23×10^{-7}	0.005**	0.016*	2.34×10^{6}± 7.14×10^{6}	3.16×10^{5} ± 1.05×10^{6}	0.019*	0.029*
**Other types of O-glycan biosynthesis**	1.83×10^{-7} ± 4.11×10^{-7}	1.19×10^{-7} ± 5.23×10^{-7}	0.005**	0.016*	2.34×10^{6} ± 7.14×10^{6}	3.16×10^{5} ± 1.05×10^{6}	0.019*	0.029*
**Parathyroid hormone synthesis, secretion and action**	4.44×10^{-9} ± 2.23×10^{-8}	1.20×10^{-7} ± 5.83×10^{-7}	0.023*	0.034*	8.90×10^{4} ± 5.87×10^{5}	1.04×10^{6} ± 4.69×10^{6}	0.023*	0.029*
**Renin secretion**	2.19×10^{-7} ± 4.45×10^{-7}	1.59×10^{-7} ± 4.98×10^{-7}	0.013*	0.026*	1.35×10^{6} ± 5.02×10^{6}	2.68×10^{5} ± 4.28×10^{5}	＜0.001***	0.002**

* P < 0.05, ** P < 0.01, *** P＜0.001.

Supplementary Table 2 Functional differences between ET and PD based on the mean abundances of KEGG pathways at level 3.

Pathways	Relative				Absolute			
	Mean±SD (ET, n=54)	Mean±SD (PD, n=67)	*P* value	FDR corrected *P* value	Mean±SD (ET, n=54)	Mean±SD (PD, n=67)	*P* value	FDR corrected *P* value
Adherens junction	$3.79\times10^{-10} \pm 2.79\times10^{-9}$	$1.76\times10^{-7} \pm 1.01\times10^{-6}$	0.004**	0.012*	$2.46\times10^{3} \pm 1.81\times10^{4}$	$3.66\times10^{5} \pm 2.50\times10^{6}$	0.005**	0.014*
Adipocytokine signaling pathway	$1.10\times10^{-3} \pm 3.64\times10^{-4}$	$9.33\times10^{-4} \pm 3.69\times10^{-4}$	0.009**	0.018*	$1.55\times10^{10} \pm 2.40\times10^{10}$	$1.27\times10^{10} \pm 3.61\times10^{10}$	0.003**	0.014*
Antifolate resistance	$3.78\times10^{-3} \pm 4.45\times10^{-4}$	$3.60\times10^{-3} \pm 4.47\times10^{-4}$	0.002**	0.008**	$4.96\times10^{10} \pm 6.57\times10^{10}$	$3.94\times10^{10} \pm 9.39\times10^{10}$	0.005**	0.014
Apoptosis	$3.43\times10^{-4} \pm 2.10\times10^{-4}$	$2.24\times10^{-4} \pm 2.28\times10^{-4}$	<0.001***	0.004**	$5.86\times10^{9} \pm 1.05\times10^{10}$	$4.94\times10^{9} \pm 1.77\times10^{10}$	<0.001***	0.014*
Apoptosis - fly	$4.45\times10^{-4} \pm 9.75\times10^{-5}$	$3.96\times10^{-4} \pm 1.05\times10^{-4}$	0.012*	0.020*	$5.72\times10^{9} \pm 7.62\times10^{9}$	$4.40\times10^{9} \pm 1.07\times10^{10}$	0.003**	0.014*
Autophagy - animal	$4.06\times10^{-9} \pm 2.23\times10^{-8}$	$7.34\times10^{-7} \pm 4.59\times10^{-6}$	0.043*	0.044*	$8.82\times10^{4} \pm 5.87\times10^{5}$	$2.49\times10^{6} \pm 1.27\times10^{7}$	0.044*	0.044*
Basal transcription factors	$1.18\times10^{-7} \pm 1.71\times10^{-7}$	$5.03\times10^{-6} \pm 2.79\times10^{-5}$	<0.001***	<0.001***	$1.15\times10^{6} \pm 4.20\times10^{6}$	$1.67\times10^{7} \pm 7.71\times10^{7}$	0.010*	0.014*

**beta-Lactam resistance**	$7.92\times10^{-3} \pm 8.54\times10^{-4}$	$7.65\times10^{-3} \pm 8.30\times10^{-4}$	0.042*	0.044*	$1.04\times10^{11} \pm 1.43\times10^{11}$	$8.23\times10^{10} \pm 2.00\times10^{11}$	0.004**	0.014*
**Biosynthesis of type II polyketide products**	$2.45\times10^{-9} \pm 8.16\times10^{-9}$	$1.83\times10^{-7} \pm 1.01\times10^{-6}$	0.009**	0.018*	$1.90\times10^{4} \pm 7.76\times10^{4}$	$6.26\times10^{5} \pm 3.24\times10^{6}$	0.012*	0.016*
**Biotin metabolism**	$6.46\times10^{-3} \pm 8.32\times10^{-4}$	$5.74\times10^{-3} \pm 9.14\times10^{-4}$	$<$ 0.001***	$<$ 0.001***	$8.67\times10^{10} \pm 1.17\times10^{11}$	$6.89\times10^{10} \pm 1.87\times10^{11}$	0.002**	0.014*
**Caffeine metabolism**	$2.27\times10^{-9} \pm 1.01\times10^{-8}$	$1.78\times10^{-7} \pm 1.01\times10^{-6}$	0.012*	0.019*	$3.38\times10^{3} \pm 1.89\times10^{4}$	$4.43\times10^{5} \pm 2.56\times10^{6}$	0.008**	0.014*
**Cationic antimicrobial peptide (CAMP) resistance**	$5.29\times10^{-3} \pm 1.39\times10^{-3}$	$4.75\times10^{-3} \pm 1.35\times10^{-3}$	0.014*	0.022*	$6.85\times10^{10} \pm 9.35\times10^{10}$	$5.62\times10^{10} \pm 1.62\times10^{11}$	0.003**	0.014*
**Cell adhesion molecules (CAMs)**	$2.84\times10^{-8} \pm 6.59\times10^{-8}$	$7.92\times10^{-8} \pm 2.21\times10^{-7}$	0.022*	0.028*	$1.64\times10^{5} \pm 4.34\times10^{5}$	$2.03\times10^{5} \pm 5.75\times10^{5}$	0.038*	0.039*
**Cell cycle**	$1.24\times10^{-7} \pm 2.39\times10^{-7}$	$1.42\times10^{-6} \pm 5.04\times10^{-6}$	0.001**	0.006**	$7.62\times10^{5} \pm 1.85\times10^{6}$	$3.61\times10^{6} \pm 1.37\times10^{7}$	0.008**	0.014*
**Cell cycle - yeast**	$1.20\times10^{-7} \pm 2.39\times10^{-7}$	$6.90\times10^{-7} \pm 2.26\times10^{-6}$	0.005**	0.013*	$6.74\times10^{5} \pm 1.73\times10^{6}$	$1.12\times10^{6} \pm 2.75\times10^{6}$	0.035*	0.036*

**Chemokine signaling pathway**	3.79×10^{-10} ± 2.79×10^{-9}	1.76×10^{-7} ± 1.01×10^{-6}	0.004**	0.012*	2.46×10^{3} ± 1.81×10^{4}	3.66×10^{5} ± 2.50×10^{6}	0.005**	0.014*
**Citrate cycle (TCA cycle)**	1.19×10^{-2} ± 1.38×10^{-3}	1.11×10^{-2} ± 1.59×10^{-3}	0.002**	0.007**	1.57×10^{11} ± 2.12×10^{11}	1.25×10^{11} ± 3.08×10^{11}	0.004**	0.014*
**Cyanoamino acid metabolism**	4.22×10^{-3} ± 7.58×10^{-4}	3.88×10^{-3} ± 7.28×10^{-4}	0.007**	0.015*	5.87×10^{10} ± 8.68×10^{10}	4.75×10^{10} ± 1.29×10^{11}	0.003**	0.014*
**D-Arginine and D-ornithine metabolism**	8.07×10^{-5} ± 6.74×10^{-5}	5.83×10^{-5} ± 6.41×10^{-5}	0.005**	0.014*	7.15×10^{8} ± 7.40×10^{8}	5.53×10^{8} ± 1.19×10^{9}	< 0.001***	0.014*
**Drug metabolism - other enzymes**	5.47×10^{-3} ± 4.24×10^{-4}	5.27×10^{-3} ± 3.96×10^{-4}	0.017*	0.024*	7.07×10^{10} ± 9.26×10^{10}	5.54×10^{10} ± 1.32×10^{11}	0.005**	0.014*
**ECM-receptor interaction**	2.84×10^{-8} ± 6.59×10^{-8}	7.92×10^{-8} ± 2.21×10^{-7}	0.022*	0.028*	1.64×10^{5} ± 4.34×10^{5}	2.03×10^{5} ± 5.75×10^{5}	0.038*	0.039*
**EGFR tyrosine kinase inhibitor resistance**	4.06×10^{-9} ± 2.23×10^{-8}	7.34×10^{-7} ± 4.59×10^{-6}	0.043*	0.044*	8.82×10^{4} ± 5.87×10^{5}	2.49×10^{6} ± 1.27×10^{7}	0.044*	0.044*
**Fanconi anemia pathway**	8.12×10^{-9} ± 4.45×10^{-8}	1.47×10^{-6} ± 9.17×10^{-6}	0.027*	0.033*	1.76×10^{5} ± 1.17×10^{6}	5.06×10^{6} ± 2.54×10^{7}	0.027*	0.029*
**Fc gamma**	7.61×10^{-10} ±	2.87×10^{-7} ± 1.17×10^{-6}	< 0.001*	0.002**	3.33×10^{3} ±	9.21×10^{5} ±	< 0.001*	0.014*

R-mediated phagocytosis	3.92×10^{-9}		**		1.91×10^{4}	4.35×10^{6}	**	
Ferroptosis	$1.28\times10^{-3}\pm4.21\times10^{-4}$	$1.05\times10^{-3}\pm4.38\times10^{-4}$	0.003**	0.009**	$1.85\times10^{10}\pm2.91\times10^{10}$	$1.52\times10^{10}\pm4.52\times10^{10}$	0.002**	0.014*
Focal adhesion	$2.84\times10^{-8}\pm6.59\times10^{-8}$	$7.92\times10^{-8}\pm2.21\times10^{-7}$	0.022*	0.028*	$1.64\times10^{5}\pm4.34\times10^{5}$	$2.03\times10^{5}\pm5.75\times10^{5}$	0.038*	0.039*
Folate biosynthesis	$9.36\times10^{-3}\pm1.23\times10^{-3}$	$8.66\times10^{-3}\pm1.10\times10^{-3}$	0.002**	0.007**	$1.22\times10^{11}\pm1.58\times10^{11}$	$9.58\times10^{10}\pm2.36\times10^{11}$	0.004**	0.014*
Glycosaminoglycan degradation	$1.67\times10^{-3}\pm8.79\times10^{-4}$	$1.30\times10^{-3}\pm8.22\times10^{-4}$	0.006**	0.014*	$2.72\times10^{10}\pm4.78\times10^{10}$	$2.32\times10^{10}\pm8.20\times10^{10}$	0.003**	0.014*
Glycosphingolipid biosynthesis - ganglio series	$9.69\times10^{-4}\pm6.50\times10^{-4}$	$6.74\times10^{-4}\pm6.42\times10^{-4}$	0.003**	0.010*	$1.73\times10^{10}\pm3.33\times10^{10}$	$1.46\times10^{10}\pm5.48\times10^{10}$	＜0.001***	0.014*
Glycosphingolipid biosynthesis - globo and isoglobo series	$1.78\times10^{-3}\pm7.66\times10^{-4}$	$1.48\times10^{-3}\pm7.26\times10^{-4}$	0.015*	0.023*	$2.80\times10^{10}\pm4.86\times10^{10}$	$2.27\times10^{10}\pm7.14\times10^{10}$	0.004**	0.014*
Glyoxylate and dicarboxylate metabolism	$1.25\times10^{-2}\pm1.25\times10^{-3}$	$1.20\times10^{-2}\pm1.17\times10^{-3}$	0.018*	0.025*	$1.61\times10^{11}\pm2.14\times10^{11}$	$1.30\times10^{11}\pm3.20\times10^{11}$	0.005**	0.014*
GnRH signaling pathway	$3.82\times10^{-10}\pm2.81\times10^{-9}$	$1.11\times10^{-7}\pm6.24\times10^{-7}$	0.008**	0.016*	$8.64\times10^{2}\pm6.35\times10^{3}$	$5.55\times10^{5}\pm3.57\times10^{6}$	0.008**	0.014*

**Human T-cell leukemia virus 1 infection**	$1.22\times10^{-8} \pm 6.68\times10^{-8}$	$2.20\times10^{-6} \pm 1.38\times10^{-5}$	0.043*	0.044*	$2.65\times10^{5} \pm 1.76\times10^{6}$	$7.47\times10^{6} \pm 3.82\times10^{7}$	0.044*	0.044*
**Isoquinoline alkaloid biosynthesis**	$8.66\times10^{-4} \pm 2.02\times10^{-4}$	$7.58\times10^{-4} \pm 1.86\times10^{-4}$	0.004**	0.012*	$1.10\times10^{10} \pm 1.43\times10^{10}$	$8.65\times10^{9} \pm 2.23\times10^{10}$	0.002**	0.014*
**Leishmaniasis**	$4.06\times10^{-9} \pm 2.23\times10^{-8}$	$7.34\times10^{-7} \pm 4.59\times10^{-6}$	0.043*	0.044*	$8.82\times10^{4} \pm 5.87\times10^{5}$	$2.49\times10^{6} \pm 1.27\times10^{7}$	0.044*	0.044*
**Lipoic acid metabolism**	$6.71\times10^{-4} \pm 1.83\times10^{-4}$	$6.04\times10^{-4} \pm 1.74\times10^{-4}$	0.014*	0.021*	$9.46\times10^{9} \pm 1.48\times10^{10}$	$7.55\times10^{9} \pm 2.13\times10^{10}$	0.005**	0.014*
**Lipopolysaccharide biosynthesis**	$9.49\times10^{-3} \pm 3.82\times10^{-3}$	$6.16\times10^{-3} \pm 3.64\times10^{-3}$	$<$ 0.001***	$<$ 0.001***	$1.18\times10^{11} \pm 1.46\times10^{11}$	$8.36\times10^{10} \pm 2.31\times10^{11}$	$<$ 0.001***	0.014*
**Lysosome**	$2.93\times10^{-3} \pm 1.88\times10^{-3}$	$2.07\times10^{-3} \pm 1.81\times10^{-3}$	0.003**	0.009**	$5.08\times10^{10} \pm 9.54\times10^{10}$	$4.53\times10^{10} \pm 1.77\times10^{11}$	0.001**	0.014*
**Mitophagy - animal**	$8.12\times10^{-9} \pm 4.45\times10^{-8}$	$1.47\times10^{-6} \pm 9.17\times10^{-6}$	0.011*	0.019*	$1.76\times10^{5} \pm 1.17\times10^{6}$	$5.03\times10^{6} \pm 2.56\times10^{7}$	0.011*	0.015*
**mRNA surveillance pathway**	$1.22\times10^{-8} \pm 6.68\times10^{-8}$	$2.20\times10^{-6} \pm 1.38\times10^{-5}$	0.043*	0.044*	$2.65\times10^{5} \pm 1.76\times10^{6}$	$7.47\times10^{6} \pm 3.82\times10^{7}$	0.044*	0.044*
**mTOR signaling pathway**	$6.77\times10^{-9} \pm 2.41\times10^{-8}$	$7.46\times10^{-7} \pm 4.58\times10^{-6}$	0.012*	0.019*	$1.22\times10^{5} \pm 6.06\times10^{5}$	$2.52\times10^{6} \pm 1.27\times10^{7}$	0.020*	0.021*

**Novobiocin biosynthesis**	$1.97\times10^{-3}\pm1.95\times10^{-4}$	$1.91\times10^{-3}\pm1.63\times10^{-4}$	0.027*	0.032*	$2.44\times10^{10}\pm3.00\times10^{10}$	$1.90\times10^{10}\pm4.11\times10^{10}$	0.005**	0.014*
**One carbon pool by folate**	$9.55\times10^{-3}\pm1.03\times10^{-3}$	$9.15\times10^{-3}\pm1.04\times10^{-3}$	0.007**	0.016*	$1.26\times10^{11}\pm1.69\times10^{11}$	$1.00\times10^{11}\pm2.43\times10^{11}$	0.005**	0.014*
**Other glycan degradation**	$5.08\times10^{-3}\pm2.65\times10^{-3}$	$3.88\times10^{-3}\pm2.61\times10^{-3}$	0.002**	0.008**	$8.47\times10^{10}\pm1.53\times10^{11}$	$7.41\times10^{10}\pm2.71\times10^{11}$	0.002**	0.014*
**Pancreatic cancer**	$3.82\times10^{-10}\pm2.81\times10^{-9}$	$1.11\times10^{-7}\pm6.24\times10^{-7}$	0.008**	0.016*	$8.64\times10^{2}\pm6.35\times10^{3}$	$5.55\times10^{5}\pm3.57\times10^{6}$	0.008**	0.014*
**Parathyroid hormone synthesis, secretion and action**	$4.44\times10^{-9}\pm2.24\times10^{-8}$	$8.77\times10^{-7}\pm4.85\times10^{-6}$	<0.001***	0.002**	$8.90\times10^{4}\pm5.87\times10^{5}$	$3.13\times10^{6}\pm1.36\times10^{7}$	<0.001***	0.014*
**Penicillin and cephalosporin biosynthesis**	$4.07\times10^{-4}\pm1.65\times10^{-4}$	$3.36\times10^{-4}\pm1.49\times10^{-4}$	0.009**	0.018*	$5.99\times10^{9}\pm9.50\times10^{9}$	$4.96\times10^{9}\pm1.56\times10^{10}$	0.003**	0.014*
**Pentose and glucuronate interconversions**	$6.09\times10^{-3}\pm1.45\times10^{-3}$	$5.47\times10^{-3}\pm1.34\times10^{-3}$	0.016*	0.024*	$8.40\times10^{10}\pm1.22\times10^{11}$	$7.02\times10^{10}\pm2.10\times10^{11}$	0.004**	0.014*
**Phagosome**	$6.77\times10^{-9}\pm2.41\times10^{-8}$	$7.46\times10^{-7}\pm4.58\times10^{-6}$	0.012*	0.019*	$1.22E\times10^{5}\pm6.06\times10^{5}$	$2.52\times10^{6}\pm1.27\times10^{7}$	0.020*	0.021*

**Phenylalanine metabolism**	3.87E$\times10^{-3}$ ± 1.18$\times10^{-3}$	3.55$\times10^{-3}$ ± 1.13$\times10^{-3}$	0.031*	0.036*	4.68$\times10^{10}$ ± 5.90$\times10^{10}$	3.87$\times10^{10}$ ± 1.06$\times10^{11}$	0.003**	0.014*
**PPAR signaling pathway**	1.86E$\times10^{-3}$ ± 4.15$\times10^{-4}$	1.64$\times10^{-3}$ ± 4.12$\times10^{-4}$	0.003**	0.010*	2.57$\times10^{10}$ ± 3.79$\times10^{10}$	2.07$\times10^{10}$ ± 5.63$\times10^{10}$	0.003**	0.014*
**Prodigiosin biosynthesis**	2.56E$\times10^{-3}$ ± 3.81$\times10^{-4}$	2.45$\times10^{-3}$ ± 3.31$\times10^{-4}$	0.022*	0.028*	3.43$\times10^{10}$ ± 4.64$\times10^{10}$	2.63$\times10^{10}$ ± 6.25$\times10^{10}$	0.005**	0.014*
**Protein digestion and absorption**	4.09E$\times10^{-4}$ ± 2.40$\times10^{-4}$	2.93$\times10^{-4}$ ± 2.60$\times10^{-4}$	0.004**	0.010*	6.96$\times10^{9}$ ± 1.29$\times10^{10}$	6.27$\times10^{9}$ ± 2.28$\times10^{10}$	0.002**	0.014*
**Ras signaling pathway**	3.82$\times10^{-10}$ ± 2.81$\times10^{-9}$	1.11$\times10^{-7}$ ± 6.24$\times10^{-7}$	0.008**	0.016*	8.64$\times10^{2}$ ± 6.35$\times10^{3}$	5.55$\times10^{5}$ ± 3.57$\times10^{6}$	0.008**	0.014*
**Regulation of actin cytoskeleton**	2.84$\times10^{-8}$ ± 6.59$\times10^{-8}$	7.92$\times10^{-8}$ ± 2.21$\times10^{-7}$	0.022*	0.028*	1.64$\times10^{5}$ ± 4.34$\times10^{5}$	2.03$\times10^{5}$ ± 5.75$\times10^{5}$	0.038*	0.039*
**Riboflavin metabolism**	4.64$\times10^{-3}$ ± 6.13$\times10^{-4}$	4.28$\times10^{-3}$ ± 5.89$\times10^{-4}$	0.001**	0.005**	5.98$\times10^{10}$ ± 7.55$\times10^{10}$	4.77$\times10^{10}$ ±	0.004**	0.014*
**Sphingolipid metabolism**	3.18$\times10^{-3}$ ± 1.18$\times10^{-3}$	2.73$\times10^{-3}$ ± 1.07$\times10^{-3}$	0.011*	0.019*	4.87$\times10^{10}$ ± 8.08$\times10^{10}$	4.00$\times10^{10}$ ±	0.004**	0.014*
**Spinocerebellar ataxia**	1.03$\times10^{-4}$ ± 7.88$\times10^{-5}$	7.03$\times10^{-5}$ ± 8.93$\times10^{-5}$	0.001**	0.006**	1.96$\times10^{9}$ ± 4.05$\times10^{9}$	1.81$\times10^{9}$ ±	$<$0.001***	0.014*
**Steroid degradation**	2.00$\times10^{-7}$ ± 3.89$\times10^{-7}$	7.28$\times10^{-7}$ ± 1.39$\times10^{-6}$	$<$0.001***	$<$0.001***	1.79$\times10^{6}$ ± 5.48$\times10^{6}$	2.25$\times10^{6}$ ±	$<$0.001***	0.014*

**Steroid hormone biosynthesis**	3.20×10^{-4} ± 2.50×10^{-4}	2.06×10^{-4} ± 1.82×10^{-4}	0.002**	0.008**	5.43×10^{9} ± 1.02×10^{10}	4.27×10^{9} ±	<0.001***	0.014*
**Synaptic vesicle cycle**	4.36×10^{-9} ± 1.38×10^{-8}	5.55×10^{-8} ± 3.38×10^{-7}	0.008**	0.016*	4.35×10^{4} ± 1.74×10^{5}	2.55×10^{5} ±	0.016*	0.018*
**Tetracycline biosynthesis**	6.43×10^{-6} ± 1.23×10^{-5}	3.13×10^{-6} ± 5.77×10^{-6}	0.010**	0.018*	1.53×10^{8} ± 6.23×10^{8}	4.11×10^{7} ±	0.002**	0.014*
**Thermogenesis**	1.06×10^{-3} ± 3.71×10^{-4}	8.78×10^{-4} ± 3.76×10^{-4}	0.006**	0.015*	1.51×10^{10} ± 2.35×10^{10}	1.23×10^{10} ±	0.003**	0.014*
**Tight junction**	4.44×10^{-9} ± 2.24×10^{-8}	9.10×10^{-7} ± 4.67×10^{-6}	<0.001***	0.004**	9.06×10^{4} ± 5.87×10^{5}	2.86×10^{6} ±	<0.001***	0.014*
**Tropane, piperidine and pyridine alkaloid biosynthesis**	1.60×10^{-3} ± 1.94×10^{-4}	1.47×10^{-3} ± 2.00×10^{-4}	<0.001	0.003**	2.01×10^{10} ± 2.53×10^{10}	1.58×10^{10} ±	0.003**	0.014*
**Various types of N-glycan biosynthesis**	9.71×10^{-4} ± 6.50×10^{-4}	6.79×10^{-4} ± 6.42×10^{-4}	0.004**	0.010*	1.73×10^{10} ± 3.33×10^{10}	1.47×10^{10} ±	0.001**	0.014*
**Vitamin B6 metabolism**	2.93×10^{-4} ± 2.38×10^{-4}	2.80×10^{-3} ± 2.98×10^{-4}	0.011*	0.019*	3.76×10^{10} ± 4.92×10^{10}	3.03×10^{10} ±	0.004**	0.014*
**Zeatin**	7.82×10^{-4} ±	7.37×10^{-4} ± 1.26×10^{-4}	0.023*	0.030*	1.06×10^{10} ±	8.44×10^{9} ±	0.005**	0.014*

**biosynthesis**	1.30×10^{-4}	1.46×10^{10}

* P < 0.05, ** P < 0.01, *** P＜0.001.

Supplementary Methods

Contents:

1. **DNA extraction**
2. **PCR amplification of target genes and nova sequencing**
3. **Quantitative real-time PCR**
4. **Bioinformatic analysis**

DNA extraction

Samples were stored at −80 ℃ until DNA extraction. The DNA was extracted from 200 mg samples using the QIAamp DNA Stool Mini Kit (QIAGEN, Hilden, Germany) following the manufacturer's instructions. DNA was checked by running the samples on 1.2% agarose gels.

PCR amplification of target genes and nova sequencing

Polymerase chain reaction (PCR) amplification of target gene was performed using general primers: 357F(5'-ACTCCTACGGRAGGCAGCAG-3') and 806R(5'-GGACTACHVGGGTWTCTAAT-3').

The primers also contained the Illumina 5'overhang adapter sequences for two-step amplicon library building, following manufacturer's instructions for the overhang sequences and barcodes. The initial PCR reactions were carried out 50 μL reaction volumes with 1-2 μL DNA template, 200 uM dNTPs, 0.2 uM of each primer, 5X reaction buffer 10uL and 1U Phusion DNA Polymerase (New England Biolabs, USA). PCR conditions consisted of initial denaturation at 94℃ for 2 min, followed by 25 cycles of denaturation at 94℃ for 30 s, annealing at 56℃ for 30 s and extension at 72℃ for 30 s, with a final extension of 72℃ for 5 min. The barcoded PCR products were purified using a DNA gel extraction kit (Axygen, USA) and quantified using the FTC -3000 TM real-time PCR（Funglyn Shanghai）. The PCR products from different samples were mixed at equal ratios. The second step PCR with dual 8bp barcodes was used for multiplexing. Eight cycle PCR reactions were used to incorporate two unique barcodes to either end of the amplicons. Cycling conditions consisted of one cycle of 94℃ for 3 min, followed by eight cycles of 94℃ for 30 s, 56℃ for 30 s and 72℃ for 30 s, followed by a final extension cycle of 72℃ for 5 min. The library was purified using a DNA gel extraction kit (Axygen, USA) and sequenced by 2*250 bp paired-end sequencing on the Novaseq platform using Novaseq 6000 SP 500 Cycle Reagent Kit (Illumina USA) at TinyGen Bio-Tech (Shanghai) Co., Ltd.

Quantitative real-time PCR

Quantitative real-time PCRs (qPCRs) were performed with a FTC-3000TM Real-Time Quantitative Thermal Cycler (Funglyn, Shanghai, China). All qPCR

reactions were run with 3 replicates per DNA. Standard curves were set up by serially diluting plasmid of a pMD18-T vector with the appropriate insert from 107 to 1012 target gene copies µl-1 for every primer set. The standard curve was obtained using linear regression of threshold cycle numbers (cT) versus log copy numbers of targets.

qPCR reactions were performed in 25 µl reaction mixtures that were composed of 12.5 µl SRBR Premix Ex TaqTM（2×） (Takara, Japan), 1 µl (10 µM) of each forward and reverse primers （357F and 806R）, 5 µl of template DNA and sterilized deionized water. Thermal protocols and primers were as described in the manuscript. Melting Curve analyses were performed from 60 to 96℃ with increments of 0.1℃ per cycle.

Bioinformatic analysis

The raw fastq files were demultiplexed based on the barcode. PE reads for all samples were run through Trimmomatic[1] (version 0.35) to remove low quality base pairs using these parameters (SLIDINGWINDOW: 50:20 MINLEN: 50). Trimmed reads were then further merged using FLASH[2] program (version 1.2.11) with default parameters. The low quality contigs were removed based on screen.seqs command using the following filtering parameters, maxambig=0, minlength = 200, maxlength =485, maxhomop= 8.

The 16S sequences were analyzed using a combination of software mothur[3] (version 1.33.3),UPARSE[4](usearch version v8.1.1756, http://drive5.com/uparse/), and R[5, 6] (version 3.6.3).

The demultiplexed reads were clustered at 97% sequence identity into operational taxonomic units (OTUs) and the singleton OTUs were deleted using the UPARSE pipeline to avoid spurious low-abundance taxa. (https://drive5.com/usearch/manual8.1/uparse_pipeline.html).

The OTU representative sequences were assignment for taxonomy against Silva 128 database with confidence score ≥ 0.6 by the classify.seqs command in mothur.

The relative abundances obtained from 16S rRNA sequencing were quantified to get absolute abundance (copies/gram) by multiplying the total bacterial load performed by qPCR.

For the alpha-diversity analysis, Shannon, Simpson, Chao1 index and rarefaction curves were calculated were using mothur and plotted by R. Kruskal-Wallis/Wilcoxon rank-sum test was used to detect the significant changes of Shannon, Simpson, Chao1 index between groups, performed with “ggpubr::compare_means” function in “ggpubr” package of R. The ridge regression analysis was then performed to confirm the confounding factors by adjusting for the significantly different clinical characteristics among groups.

For the beta-diversity metrics, the weighted UniFrac distance matrix were visualized with Principal Coordinate Analysis (PCoA) by ape package in R. Analysis

of Similarity (ANOSIM)[6] was performed with “vegan” package of R, based on Weighted.unifrac distance, to compare within- and between-group similarity. To test for confounding, permutational multivariate analysis of variance (PERMANOVA)[7] with groups and significantly different charactersistics as covariates was performed with “adonis” function in “vegan” package of R with 9,999 permutations and the fraction of the total variance explained by each variable was calculated in this model.

LEfSE[7] analysis was used to identify taxa significantly enriched in the essential tremor (ET) group compared with control group (NC) or Parkinson’s disease (PD), as weel as between NC and PD, using the persample normalization value of 1,000,000 and default values for other parameters. In LEfSE analysis, the linear discriminant analysis (LDA) score was computed for taxa differentially abundant between the two groups. A taxon at $P < 0.05$ (Kruskal–Wallis test) and log10[LDA]≥ 2.0 was considered significant. The area under the curve (AUC) and the diagnostic performances of 4 most discriminant genera between groups with the highest LDA scores extracted from the consistent results (overlapped genera) of relative and absolute data were obtained with receiver operating characteristics (ROC) curves analysis.

Microbiota predicted functional profiling: Inferred functional profiling of the microbiota was performed by PICRUSt2[8] (v2.3.0, https://github.com/picrust/picrust2) using the picrust2_pipeline.py command. The input file was OTU representative sequence and .biom feature table file. For each pathway, the data takes the form of “relative abundance” and “absolute abundance”, defined as the proportion of that pathway over the sum of total pathways for each sample.Wilcoxon rank sum test is performed to compared the differential metabolic pathway.

Spearman rank-correlation analysis was applied to explore the relation between the clinical features and specific microbiota taxa in ET. The ridge regression analysis was then performed to confirm the correlation effect, adjusting for sex, age, BMI, smoking, alcohol drinking, coffee drinking, tea drinking and diabetes. $P < 0.05$ was considered as statistically significant.

References

[1] Bolger, A.M., Lohse, M. & Usadel, B. Trimmomatic: a flexible trimmer for Illumina sequence data. *Bioinformatics.* **30**, 2114-2120 (2014).

[2] Magoč, T. & Salzberg, S.L. FLASH: fast length adjustment of short reads to improve genome assemblies. *Bioinformatics.* **27**, 2957-2963 (2011).

[3] Schloss, P.D. et al. Introducing mothur: open-source, platform-independent, community-supported software for describing and comparing microbial communities. *Appl Environ Microbiol.* **75**, 7537-7541(2009).

[4] Alloui,T. et al. Usearch: A Meta Search Engine based on a new result merging strategy. 2015 7th International Joint Conference on Knowledge Discovery, Knowledge Engineering and Knowledge Management (IC3K), 531-536 (2015).

[5] Ihaka, R & Gentleman, R.R. A Language for Data Analysis and Graphics. *J Comput Graph Stat*. **4**, 299-314 (1996).

[6] Clarke K.R. Non-parametric multivariate analyses of changes in community structure. *Austral Ecol*. **18**, 117–143 (1993).

[7] Anderson, M.J. A new method for non-parametric multivariate analysis of variance. *Austral Ecol.* **26**, 32-46 (2001).

[8] Segata, N. et al. Metagenomic biomarker discovery and explanation. *Genome Biol.* **12**, R60 (2011).

[9] Douglas G.M. et al. PICRUSt2: An improved and customizable approach for metagenome inference. Posted online. http://doi.org/10.1101/672295 (2020).
